# Minocycline Treatment Reverses Sound Evoked EEG Abnormalities in a Mouse Model of Fragile X Syndrome

**DOI:** 10.3389/fnins.2020.00771

**Published:** 2020-08-04

**Authors:** Jonathan W. Lovelace, Iryna M. Ethell, Devin K. Binder, Khaleel A. Razak

**Affiliations:** ^1^Department of Psychology and Neuroscience Graduate Program, University of California, Riverside, Riverside, CA, United States; ^2^Neuroscience Graduate Program, University of California, Riverside, Riverside, CA, United States; ^3^Division of Biomedical Sciences, School of Medicine, University of California, Riverside, Riverside, CA, United States

**Keywords:** fragile X syndrome, autism, forebrain, sensory hypersensitivity, EEG, minocycline, MMP-9

## Abstract

Fragile X Syndrome (FXS) is a leading known genetic cause of intellectual disability. Many symptoms of FXS overlap with those in autism including repetitive behaviors, language delays, anxiety, social impairments and sensory processing deficits. Electroencephalogram (EEG) recordings from humans with FXS and an animal model, the *Fmr1* knockout (KO) mouse, show remarkably similar phenotypes suggesting that EEG phenotypes can serve as biomarkers for developing treatments. This includes enhanced resting gamma band power and sound evoked total power, and reduced fidelity of temporal processing and habituation of responses to repeated sounds. Given the therapeutic potential of the antibiotic minocycline in humans with FXS and animal models, it is important to determine sensitivity and selectivity of EEG responses to minocycline. Therefore, in this study, we examined if a 10-day treatment of adult *Fmr1* KO mice with minocycline (oral gavage, 30 mg/kg per day) would reduce EEG abnormalities. We tested if minocycline treatment has specific effects based on the EEG measurement type (e.g., resting versus sound-evoked) from the frontal and auditory cortex of the *Fmr1* KO mice. We show increased resting EEG gamma power and reduced phase locking to time varying stimuli as well as the 40 Hz auditory steady state response in the *Fmr1* KO mice in the pre-drug condition. Minocycline treatment increased gamma band phase locking in response to auditory stimuli, and reduced sound-evoked power of auditory event related potentials (ERP) in *Fmr1* KO mice compared to vehicle treatment. Minocycline reduced resting EEG gamma power in *Fmr1* KO mice, but this effect was similar to vehicle treatment. We also report frequency band-specific effects on EEG responses. Taken together, these data indicate that sound-evoked EEG responses may serve as more sensitive measures, compared to resting EEG measures, to isolate minocycline effects from placebo in humans with FXS. Given the use of minocycline and EEG recordings in a number of neurodegenerative and neurodevelopmental conditions, these findings may be more broadly applicable in translational neuroscience.

## Introduction

Fragile X Syndrome (FXS) is a genetic cause of intellectual disability (Crawford et al., 2001) that results from a mutation in the *Fragile X Mental Retardation 1 (Fmr1)* gene and down-regulation of Fragile X Mental Retardation Protein (FMRP) (Yu et al., 1991). FMRP is an RNA binding protein, and one of the fundamental cellular effects of FMRP loss is abnormal protein synthesis including proteins that regulate synaptic function (Darnell et al., 2011). FXS symptoms include intellectual disability, anxiety, repetitive behaviors, social communication deficits, delayed language development and abnormal sensory processing (Wisniewski et al., 1991; Abbeduto and Hagerman, 1997; Miller et al., 1999; Musumeci et al., 1999; Roberts et al., 2001; Sabaratnam et al., 2001; Berry-Kravis, 2002; Hagerman and Hagerman, 2002; Hagerman et al., 2009; Van der Molen et al., 2010; Sinclair et al., 2017b). Diagnosis of autism in children with FXS ranges between 18–30% (Rogers et al., 2001; Belmonte and Bourgeron, 2006). The overlap in symptoms of FXS and autism suggest overlapping cellular and circuit mechanisms.

Abnormal sensory processing in humans with FXS includes hypersensitivity and reduced habituation to repeated sensory stimuli (Castrén et al., 2003; Schneider et al., 2013). These symptoms affect multiple sensory systems (Rais et al., 2018), are seen from early in development, and may lead to cognitive deficits and anxiety (Orefice et al., 2016). Both humans with FXS (Castrén et al., 2003; Schneider et al., 2013; Van der Molen and Van der Molen, 2013; Ethridge et al., 2016) and *Fmr1* knockout (KO) mice (Rotschafer and Razak, 2013, 2014; Lovelace et al., 2016; McCullagh et al., 2020) show debilitating auditory hypersensitivity. Recent EEG recordings from humans show altered cortical oscillatory activity in FXS. Wang et al. (2017) compared age-matched healthy controls (mean age = 26.4 years) and FXS patients (mean age = 25.6) and showed enhanced resting state gamma frequency power and greater spatial spread of phase-synchronized gamma band activity in FXS patients that may mark abnormally activated cortex and hypersensitivity (Merker, 2013). When neural oscillations were induced with sounds, inter-trial phase coherence (phase locking factor) was reduced in FXS patients, particularly at gamma frequencies (Ethridge et al., 2017, 2019). Van der Molen et al. (2012) studied age-matched control and FXS patients (mean age = 29 years) and showed enhanced N1 amplitude of the ERPs in response to tones. These results, consistent with Castrén et al. (2003), indicate abnormally enhanced tone evoked responses and/or enhanced synchrony of responding population of cells. Ethridge et al. (2016) studied age-matched control (mean age = 28.8 years) and patients with FXS (mean age = 28.5) and recorded ERPs in response to repetitions (2 Hz repetition rate) of the same tone (1000 Hz, 65 dB). They found that the N1 amplitude showed reduced habituation across repeated stimuli in humans with FXS. They also reported decreased phase locking and increased single-trial power in the gamma band. The phase locking deficits in the gamma range suggest an inability of underlying circuits to generate appropriately timed inhibitory responses. Importantly, the EEG phenotypes were correlated with abnormal sensory sensitivity reports and deficits in social communication as measured with the social and communication questionnaire. These correlations suggest clinical significance of EEG phenotypes in humans with FXS.

Remarkably similar EEG differences are observed in *Fmr1* KO, compared to wild type (WT), mice. Lovelace et al. (2018) showed enhanced resting EEG gamma band power, reduced sound evoked inter-trial phase coherence and increased single trial gamma power in both auditory and frontal cortex (AC, FC) of *Fmr1* KO mice. Increased amplitude and reduced habituation of auditory ERPs are also seen in the *Fmr1* KO mice (Lovelace et al., 2016; Sinclair et al., 2017b; Wen et al., 2019; Kulinich et al., 2020). These data are consistent with *in vivo* cortical single cell recordings that show auditory hyper-responsiveness and broader frequency tuning curves (Rotschafer and Razak, 2013; Wen et al., 2018) and *in vitro* cortical slice preparations that show enhanced gamma band power in auditory cortex and synchrony across cortical layers (Goswami et al., 2019). Sinclair et al. (2017a) showed that racemic baclofen, an agonist of GABA-b receptors, reduced abnormalities in sound evoked gamma oscillations in the ERP recorded from *Fmr1* KO mice. Kulinich et al. (2020) showed that controlled sound exposure during early development reduced ERP N1 amplitude in adult Fmr1 KO mice. These data indicate that ERP parameters are sensitive to drug treatment and developmental acoustic experience. The similarities in human and mouse EEG phenotypes suggest multiple translation relevant and objective biomarkers that could be used for both development of treatments and to understand cellular mechanisms of drug action in FXS.

One promising therapeutic pathway to reduce sensory hypersensitivity in FXS patients is the reduction of matrix metalloprotease-9 (MMP-9). MMP-9 is an endopeptidase involved in activity-dependent modifications of the extracellular matrix (Reinhard et al., 2015). MMP-9 is a target of FMRP-dependent translation regulation, and MMP-9 activity is upregulated in the auditory cortex (Wen et al., 2018) and inferior colliculus (Kokash et al., 2019) of *Fmr1* KO mice. Genetic reduction of MMP-9 ameliorated correlates of auditory hypersensitivity in the *Fmr1* KO mice including normalization of auditory ERP habituation (Lovelace et al., 2016), pre-pulse inhibition of auditory startle (Kokash et al., 2019) and calling rate of ultrasonic vocalizations (Toledo et al., 2019). Single cell hyper-responsiveness and abnormal development of parvalbumin (PV) expressing inhibitory neurons in the auditory cortex of *Fmr1* KO mice were also reversed by genetic reduction of MMP-9 in *Fmr1* KO mice (Wen et al., 2018). These data suggest that treatments that reduce MMP-9 may be effective in reducing sensory symptoms in FXS. Consistent with this notion, minocycline has received considerable attention in treating FXS symptoms. Minocycline is a tetracycline antibiotic with multiple targets of action that include inflammation, apoptosis and microglial clearance. Minocycline also reduces MMP-9 levels (Dziembowska et al., 2013), and this effect may be associated with beneficial effects of minocycline treatment on neurobehavioral symptoms in multiple studies of animal models (Bilousova et al., 2009; Siller and Broadie, 2011; Rotschafer et al., 2012; Dansie et al., 2013; Toledo et al., 2019). These improvements include improvements in anxiety-like behavioral outcomes, ultrasonic vocalizations, pre-pulse inhibition of startle, dendritic spine density measurements and audiogenic seizures. Changes to MMP-9 with minocycline may also underlie beneficial effects reported in humans with FXS (Paribello et al., 2010; Leigh et al., 2013; Schneider et al., 2013). Paribello et al. (2010) conducted an open label study of minocycline treatment on FXS patients (mean age = 18 years) and found improvements in the irritability subscale of the Aberrant Behavior Checklist-Community, the Clinical Global Improvement Scale and the Visual Analog Scale for behavior. Benefits were seen in both adolescents and adults. Leigh et al. (2013) conducted a randomized, placebo controlled, double blind study of minocycline treatment (3 months) in children and adolescents with FXS (mean age = 9.2 years). They also found an improvement in the Clinical Global Improvement Scale and in the mood-related behaviors on the Visual Analog Scale (minocycline 5.26 ± 0.46 cm, placebo 4.05 ± 0.46 cm; p 0.0488).

Despite the evidence for beneficial effects of minocycline in FXS, how EEG responses are impacted by treatment is less clear. Only one study, done in humans with FXS, showed that minocycline treatment alters EEG responses. Schneider et al. (2013) used a double-blind, placebo controlled study of the effects of minocycline treatment in children (mean age = 10.5 years) with FXS. They showed that 3 months of minocycline treatment reduced N1 and P2 amplitudes and increased ERP N1 habituation to repeated tone presentation in humans with FXS, compared to placebo. But whether resting EEGs are differentially impacted by treatment compared to sound-evoked responses is not known in either mice or humans. Within resting and evoked EEGs, whether different oscillation frequencies are differentially impacted is also not known. These data are crucial to move EEG responses forward as translation-relevant outcome measures. Given the impact of MMP-9 on extracellular matrix and the functionality of parvalbumin positive inhibitory interneurons, we hypothesized that minocycline treatment of adult *Fmr1* KO mice would reduce resting state gamma power, improve gamma band phase locking and reduce ERP abnormalities, compared to vehicle treatment. Our data replicates a number of EEG/ERP phenotypes reported previously (Lovelace et al., 2018) and adds evidence for decreased temporal fidelity of 40 Hz auditory steady state responses (ASSR) in the *Fmr1* KO mice. We report that minocycline treatment reduces sound evoked, but not resting state, EEG abnormalities in *Fmr1* KO mice compared to vehicle treatment.

## Materials and Methods

### Mice

Male C57BL/6 *Fmr1* KO mice (B6.129P2-*Fmr1*^*tm*1*Cgr*^/J, stock #003025) and congenic male WT mice (stock # 000664) were obtained from Jackson Laboratories. All mice were housed in our vivarium for at least 72 h before experiments were carried out. PCR analysis of genomic DNA isolated from mouse tails were used to confirm genotypes of all the mice in this study. Mice were maintained in an AAALAC-accredited vivarium under 12-h light/dark cycles. Water and standard mouse chow were provided for *ad libitum* consumption. NIH guidelines were followed for all procedures with Institutional Animal Care and Use Committee approval of animal use procedures. EEG recordings were obtained from 21 WT and 23 *Fmr1* KO mice, with ages between postnatal day (P) 44 and P80. A 2 × 2 (genotype × treatment) experimental design was used with 12 *Fmr1* KO and 9 WT mice receiving minocycline treatment and the remaining mice receiving vehicle.

### Overview of Experimental Procedures

**FIGURE 1:**
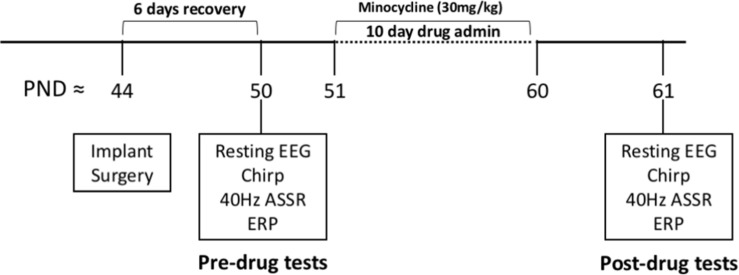
Overview of experimental procedures. All experiments were conducted on adult male C57BL/6 mice. After surgical implantation of electrodes, mice were given 6 full days to recover before EEG experiments. All mice were recorded on all EEG protocols pre-drug administration. A total of 23 *Fmr1* KO mice and 21 WT mice were tested in “pre” group before any drug administration. The mice then received either minocycline (12 KO and 9 WT) or vehicle (11 KO and 12 WT) solution through oral gavage daily for 10 days followed by “post” tests. EEG recordings were never obtained on the same day that oral gavage was administered.

Figure 1 provides an overview of the procedure. After 6 days of recovery from surgery to implant the EEG recording electrodes, EEG responses were recorded from both auditory and frontal cortex (AC, FC). These recordings served as pre-drug baseline responses to obtain genotype specific differences. After 10 days of daily minocycline (30 mg/kg) or vehicle treatment, the same mice were recorded for post drug/vehicle effects. The minocycline dose was chosen based on Bilousova et al. (2009), who showed improvement in spine maturation and performance on behavioral tests in the Fmr1 KO mice. Dansie et al. (2013) and Toledo et al. (2019) used the same dose and found improvements in hyperactivity and ultrasonic vocalization phenotypes in the *Fmr1* KO mice.

### Surgery for EEG Electrode Implantation

Surgical procedures were similar to those used in Lovelace et al. (2018, 2019), and are only briefly described here. Following a brief period of isoflurane exposure (0.2–0.5%), mice were given an i.p. injection of ketamine and xylazine (K/X, 80/10 mg/kg). Mice were secured in a bite bar and placed on a stereotaxic apparatus (model 930; Kopf, CA, United States). Anesthetic plane was monitored through periodic (10 min interval) inspection of the toe pinch reflex and Supplementary doses of K/X was administered if required. Once a stable anesthetic plane was achieved, a midline sagittal incision was made along the scalp to expose the skull. Small holes (1 mm diameter) were drilled (Foredom dental drill) in the skull overlying the right auditory cortex (−1.6 mm, +4.8 mm), right frontal lobe (+3.0 mm, +1.6 mm), and left occipital (−4.8 mm, −2.95 mm) (coordinates relative to Bregma: anterior/posterior, medial/lateral). Three channel electrode posts (Plastics One, MS333-2-A-SPC) were attached to 1 mm stainless steel screws (Plastics One, 00-96 X1/16). The screws were advanced into the skull holes until secure and to the point of contact with the dura. Dental cement was applied to additionally secure the screws to the skull. Triple antibiotic ointment was applied along the edges of the dental cement. Mice were placed on a heating pad for recovery after a subcutaneous injection of Buprenorphine (0.1 mg/kg). A second Buprenorphine injection was administered between 6 and 10 h after surgery. Mice were then singly housed and monitored daily in the vivarium until the day of first EEG recordings, 6 days after surgery. At least 4 days separated last injection of Buprenorphine and EEG recordings.

### Acoustic Stimulation for EEG Recordings

EEG recordings were done using similar procedures as Lovelace et al. (2018, 2019). Recordings were obtained in a sound-attenuated chamber lined with anechoic foam (Gretch-Ken Industries, Oregon). Sound stimuli were generated using RPvdsEx software and RZ6 hardware (Tucker Davis Technologies, FL, United States) and presented through a free-field speaker (MF1, Tucker-Davis Technologies, FL, United States) located directly above the cage (12 inches). The speaker output was ∼70 dB SPL at the floor of the recording chamber with fluctuation of +/−3 dB for frequencies between 5 and 35 kHz as measured using a 1/4 inch B&K microphone.

To quantify the ability of neural generators to produce synchronized oscillations to time varying stimuli, we employed two types of auditory stimuli. The first type of stimulation is called the auditory chirp modulated sound (henceforth, “chirp”). The chirp is a tone or noise whose amplitude is modulated using a sinusoid with increasing or decreasing frequency in the 1-100 Hz range (Artieda et al., 2004; Purcell et al., 2004; Pérez-Alcázar et al., 2008). The chirp facilitates a rapid measurement of evoked phase synchrony to auditory stimuli of varying frequencies and can be used to compare temporal processing in different groups in clinical and pre-clinical settings (e.g., Purcell et al., 2004). Ethridge et al. (2017) used a chirp to induce synchronized oscillations in their EEG recordings and identified a gamma synchronization deficit in FXS. Inter-trial phase coherence analysis (ITPC, Tallon-Baudry et al., 1996) can then be used to determine the fidelity with which neural generators synchronize oscillations to the stimulus frequencies on a trial-by-trial basis. Both humans with FXS and the *Fmr1* KO mice show deficits in phase locking factor in the gamma band frequencies (∼40 Hz) (Ethridge et al., 2017; Lovelace et al., 2018).

We used a chirp stimulus to evaluate phase synchronization in the mouse EEG recordings to identify the effects of minocycline treatment. The chirp stimulus was a 2 s duration broadband noise stimulus that was amplitude modulated (100% depth) by a sinusoid whose frequency increased from 1 to 100 Hz (up-chirp). We only assessed an up-chirps in this study since no differences were found across chirp directions in our previous work (Lovelace et al., 2018). To avoid the influence of onset responses in our calculation of ITPC to the chirp, we ramped the stimulus in sound level from 0 to 100% over 1 s (rise time) which then smoothly transitioned into the chirp. Trains of chirps were repeated 300 times, with the interval between each train randomly generated to be between 1 and 1.5 s.

Second, we used a click train to assess the auditory steady state response (ASSR). The purpose of this type of stimulus is to drive steady brain oscillations at only one specific frequency of interest. The ASSR stimulus trains consisted of 0.5 ms clicks repeated at a rate of 40 Hz over 1 s period. Each train was presented 200 times with an inter-train interval of 2 s. ASSR is a widely used diagnostic biomarker for disorders like schizophrenia (O’Donnell et al., 2013). The ASSR facilitates an assessment of the function of parvalbumin (PV) positive (PV+) GABAergic cells at their resonance frequency (∼40 Hz). Abnormal PV cell function is central to our working hypothesis on mechanisms of hyperactivity and the mechanistic action of minocycline through the inhibition of MMP-9. In addition to the chirp responses and ASSR, we recorded ERPs with broadband noise stimulus trains. Each train consisted of 10 repetitions (1 or 2 Hz repetition rate) of broadband noise, with 100 trains being presented at each repetition rate. Each noise burst was presented at 70 dB SPL and was 100 ms in duration, including a 5 ms rise/fall time. The interval between each stimulus train was 8 s.

### Electrophysiology

The BioPac system (BIOPAC Systems, Inc) was used for resting and sound evoked EEG signal acquisition from awake mice inside a custom-built Faraday cage. A 15 min habituation period was provided to each mouse when first placed in the EEG recording chamber before being connected to the BioPac system. Under brief isoflurane anesthesia, a three-channel tether was connected to the three-channel electrode post (implanted in the mouse during surgery). This tether was connected to a commutator located above the cage. An additional 15 min of habituation to being connected to the tether was provided before EEG recordings commenced.

The BioPac MP150 acquisition system was connected to two EEG 100C amplifier units (one for each channel) to which the commutator was attached. The lead to the occipital cortex was used as reference for both FC and AC screw electrodes. Signals were recorded with filters set to high-pass (>0.5 Hz) and low-pass (<100 Hz). The gain was maintained the same (10,000×) between all recordings. Data were sampled at 2.5 kHz (Acqknowledge software) and down sampled to 1024 Hz post hoc using Analyzer 2.2 (Brain Vision Inc.). TTL pulses were used to synchronize stimulus onset in each train with EEG recording. Five minutes of resting EEG was recorded first. Resting EEG is defined as recordings obtained with no specific stimuli. This was followed by ERP recordings in response to trains of broadband noise, chirps and ASSR.

### Drug and Vehicle Treatment Procedures

Drug solutions were prepared using a vehicle of 0.9% NaCl + 0.3% Tween 80, targeting an administration volume of 6 mL/kg and minocycline concentration of 30 mg/kg. Stock solutions of minocycline (MP Biomedicals) were prepared regularly and kept frozen until time of use, when they were thawed, vortexed, and then administered. All drugs and vehicle control solutions were administered daily for 10 days through a stainless steel curved oral gavage tip (Cadence Science, product #7910). After each use, gavage tips were soaked in ethanol, rinsed with DI water and autoclaved before being used again.

### Data Analysis

Data files extracted from Acqknowledge software were saved in a format compatible with Analyzer 2.2 (Brain Vision Inc). The resting EEG recordings were notch filtered at 60 Hz to remove any residual line frequency power. A semi-automatic procedure implemented in Analyzer 2.2 was used for artifact rejection after visual inspection of all EEG files. Less than 20% of data were rejected due to artifacts from any single animal recording.

The resting EEG data were divided into 2 s segments and each segment was subjected to Fast Fourier Transforms (FFT) analysis using a 10% Hanning window at 0.5 Hz bin resolution. The average power density (μV^2^/Hz) was calculated for each mouse from 1 to 100 Hz. Power was binned according to spectral bands: Delta (1–4 Hz), Theta (4–8 Hz), Alpha (8–13 Hz), Beta (13–30 Hz), Low Gamma (30–55 Hz), and High Gamma (65–100 Hz). Chirp, ASSR, and ERP traces were processed with Morlet wavelets linearly spaced from 1 to 100 Hz using voltage (μV). Wavelet coefficients were exported as complex values for use with Inter Trial Phase Coherence (ITPC) analysis. Wavelets were run with a Morlet parameter of 10. To measure phase synchronization at each frequency across trials, ITPC was calculated as follows:

$$\text{ITPC}\left(f,t\right)=\frac{1}{n}\sum_{k=1}^{n}\frac{F_{k}\left(f,t\right)}{\left|F_{k}\left(f,t\right)\right|}$$

where *f* is the frequency, *t* is the time point, and *k* is trial number. Thus, *F_*k*_(f,t)* refers to the complex wavelet coefficient at a given frequency and time for the *k*th trial.

We also measured baseline corrected single trial power for ERP analysis. Real values of spectral power (μV^2^) were derived from Morlet wavelets. Baseline correction was done by taking the average power from −250 ms through −150 ms of the ERP window for each frequency layer and directly subtracting that average power from all values for their respective frequency layer in the window on a trial by trial basis. After baseline was corrected for each trial, the trials were averaged together. This is done to isolate changes in sound evoked power from background.

### Statistical Analysis

Statistical group comparisons of ITPC and baseline corrected single trial power for the various stimulation paradigms were quantified using a Monte Carlo permutation approach. The details of the analysis were similar to Lovelace et al. (2018, 2019). Time-frequency analysis was conducted by binning time into 256 parts and frequency into 100 parts, resulting in a 100 × 256 matrix. Non-parametric analysis was used to determine contiguous regions in the matrix that were significantly different from a distribution of 2000 randomized Monte Carlo permutations based on previously published methods (Maris and Oostenveld, 2007). If the cluster sizes of the real genotype assignments (both positive and negative direction, resulting in a two-tailed alpha of *p* = 0.025) were larger than 97.25% of the random group assignments, those clusters were considered significantly different between experimental conditions.

*Fmr1* KO mice are hyperactive compared to WT mice (Dansie et al., 2013) and movement can alter cortical gain (Niell and Stryker, 2010; Fu et al., 2015). To determine if movement was a factor in creating genotype differences in power spectral density, a piezoelectric transducer was placed underneath the recording cage to detect when a mouse was moving during EEG recordings. The term “resting” refers to EEGs recorded without any specific stimuli. The term “still” is used to describe resting EEG when the mouse was stationary. The term “moving” is used to describe resting EEG when the mouse was moving based on a threshold criterion for the piezoelectric signal. Movement was confirmed by analyzing the video recording (under IR light) taken throughout the EEG recording procedure. The differences between experimental conditions in resting power were analyzed on five dependent variables using one-way MANCOVA with movement as the covariate. The independent variables were genotype + drug condition and the dependent variables were five frequency bins (theta to high gamma). A 2 × 2 × 2 design was used Genotype (WT or KO) × Drug (minocycline or vehicle) × Pre/Post. Three main comparisons were done: (1) comparing WT to KO in the “Pre” condition to establish phenotypes between genotypes; (2) comparing WT (vehicle) to KO (minocycline) to assess the level of rescue minocycline treatment had on KO mice compared to controls; and (3) comparing KO (vehicle) to KO (minocycline) to determine if minocycline had an effect that was greater than vehicle alone in KO mice. The proportion of time spent moving during the 5 min recording session was used as a covariate to identify genotype effects and control for potential effects of hyperactivity on cortical responses. Genotype comparisons were corrected for multiple comparisons using Bonferroni adjustments. Bonferroni adjusted *p*-values are reported by multiplying the *p*-value for each pairwise comparison by five (for five frequency bands), so all reported *p*-values can be interpreted using a *p* < 0.05 cutoff, while maintaining an effective α = 0.01 for each multiple comparison. If data distributions did not meet assumptions of normality or homogeneity of variance, a log_10_ transform was applied to the data to be compared. Plots and figures that show measures of power are in their untransformed original format, but all statistics reporting is done on original or transformed data that meets parametric assumptions of MANCOVA.

## Results

The main goal of this study was to determine if 10 days of daily minocycline treatment, compared to vehicle, reversed resting state and sound-evoked EEG phenotypes in the auditory and frontal cortex (AC, FC) of adult *Fmr1* KO mice. In addition, we quantified how different oscillation bands in EEG responses were affected by the treatment.

### Minocycline and Vehicle Treatment Reduced Resting State Gamma Power in *Fmr1* KO Mice

**FIGURE 2:**
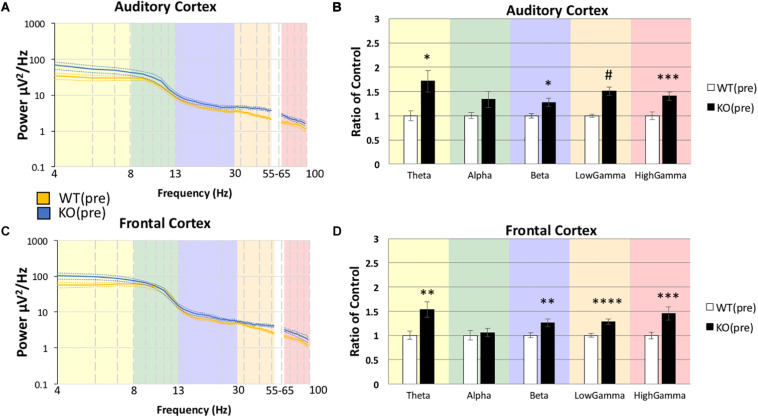
Increased resting power was observed in *Fmr1* KO mice compared to WT mice in the pre-drug condition. After mice were habituated to the recording area, 5 min resting EEG was recorded in the absence of any overt sensory stimulation. Mean power density was calculated for both genotypes using both auditory and frontal cortex electrodes **(A,C)**. For statistical comparisons, average power in each frequency band (color) was compared using standard frequency cutoffs **(B, D)**. Statistical comparisons are the result of MANCOVA, using the % movement for individual animals as a covariate, in order to isolate genotype effects from potential hyperactivity phenotypes. In general, *Fmr1* KO mice showed an increase in raw power, but the most statistically robust differences were in the low and high gamma band ranges. *p*-values were corrected for multiple comparisons using Bonferroni procedures, and each brain region was run separately (23 *Fmr1* KO mice and 21 WT mice; *p < 0.05, **p < 0.01, ***p < 0.001, ^#^*p* < 0.00001).

Before EEG recordings were made, mice were allowed to habituate to the recording chamber as well as being attached to the recording tether for a total of ∼30 min. After mice were habituated, 5 min of resting EEG was recorded in the absence of any specific auditory stimulation. Movement state was monitored by a piezo electric transducer located on the floor of the recording chamber. Power spectral density was calculated for both genotypes in the pre drug condition and in both AC and FC (Figures 2A,C). For statistical comparisons, average power in each frequency band was compared using canonical frequency cutoffs (Figures 2B,D). Statistical comparisons are the result of MANCOVA, using percentage movement for individual mice as a covariate, in order to isolate genotype and drug effects from hyperactivity phenotypes. Assumptions of equality of covariance were confirmed using Box’s M, *p* = 0.274, and Levene’s test of equality of error variances. Error variance was not different across genotypes (all *p* > 0.05). We report an effect of genotype (Pillai’s Trace = 0.646, *p* = 1.6308 × 10^–7^) across all 5 of the combined frequency variables, which include movement as a covariate. We then determined that the only frequency band that was not different between genotypes in the AC of *Fmr1* KO mice was in the alpha range, *F*(1,41) = 6.505, *p* = 0.0730, η^2^ = 0.137 (Figure 2B). All other frequency bands in the AC were significantly increased in *Fmr1* KO mice: Theta *F*(1,41) = 9.485, *p* = 0.0185, η^2^ = 0.188, Beta *F*(1,41) = 8.551, *p* = 0.0280, η^2^ = 0.173, Low Gamma *F*(1,41) = 33.366, *p* = 4.4988 × 10^–6^, η^2^ = 0.449, High Gamma *F*(1,41) = 17.156, *p* = 0.0008, η^2^ = 0.295.

The same pattern of differences were also observed in the FC: Box’s M, *p* = 0.497, Levene’s, all *p* > 0.05, Pillai’s Trace = 0.753, *p* = 4.4855 × 10^–5^), with no observed difference in alpha *F*(1,41) = 6.570, *p* = 0.0707, η^2^ = 0.138, and significant differences in all other bands, Theta *F*(1,41) = 13.657, *p* = 0.0032, η^2^ = 250, Beta *F*(1,41) = 16.085, *p* = 0.0013, η^2^ = 0.282, Low Gamma *F*(1,41) = 23.240, *p* = 9.9619 × 10^–5^, η^2^ = 0.362, High Gamma *F*(1,41) = 21.727, *p* = 0.0002, η^2^ = 0.346. In general, *Fmr1* KO mice showed an increase in power, but the most statistically robust differences were in the Gamma range. Significant differences in the gamma ranges replicate our original report (Lovelace et al., 2018) in this new cohort of mice, with a very similar “U” shape across the frequency profile.

**FIGURE 3:**
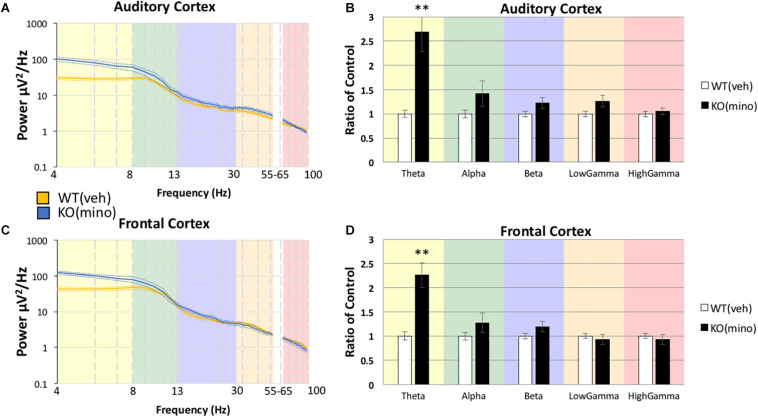
Minocycline treatment reduces resting gamma power in *Fmr1* KO mice to WT levels. After *Fmr1* KO mice were treated for 10 days with minocycline, mean power density was calculated for both groups using both auditory and frontal cortex electrodes **(A,C)**. The most significant phenotypes in the gamma frequency range were normalized and resting gamma power was similar to WT (Veh) treatment group **(B,D)**. Lower frequency deficits were still present (12 minocycline KO and 12 vehicle WT; *p < 0.05, ***p < 0.01, ****p < 0.001, *****p < 0.0001, ^#^*p* < 0.00001).

Using the same statistical approach as described above, we tested if the abnormally high resting EEG power in *Fmr1* KO mice (Figure 2) was responsive to minocycline treatment. Minocycline treated *Fmr1* KO mice were compared to vehicle treated WT mice (Figure 3) using MANCOVA in the AC (Figure 3B): Box’s M, *p* = 0.032, Levene’s, all *p* > 0.05, Pillai’s Trace = 0.789, *p* = 6.0748 × 10^–5^), with the only observed difference in Theta *F*(1,20) = 19.906, *p* = 0.0012, η^2^ = 0.499, and no significant differences in all other bands, Alpha *F*(1,20) = 1.485, *p* = 1.1858, η^2^ = 0.069, Beta *F*(1,20) = 2.931, *p* = 0.5118, η^2^ = 0.128, Low Gamma *F*(1,20) = 6.599, *p* = 0.0916, η^2^ = 0.248, High Gamma *F*(1,20) = 0.740, *p* = 1.999, η^2^ = 0.036.

The same pattern was also observed in the FC (Figure 3D): Box’s M, *p* = 0.219, Levene’s, all *p* > 0.05, Pillai’s Trace = 0.662, *p* = 0.0021), with the only observed difference in Theta *F*(1,20) = 17.589, *p* = 0.0022, η^2^ = 0.468, and no significant differences in all other bands, Alpha *F*(1,20) = 0.787, *p* = 1.927, η^2^ = 0.038, Beta *F*(1,20) = 1.477, *p* = 1.1920, η^2^ = 0.069, Low Gamma *F*(1,20) = 0.182, *p* = 3.3711, η^2^ = 0.009, High Gamma *F*(1,20) = 0.754, *p* = 1.977, η^2^ = 0.036. There were no significant differences in the gamma band power in either AC or FC, but theta power remained elevated even after minocycline treatment.

**FIGURE 4:**
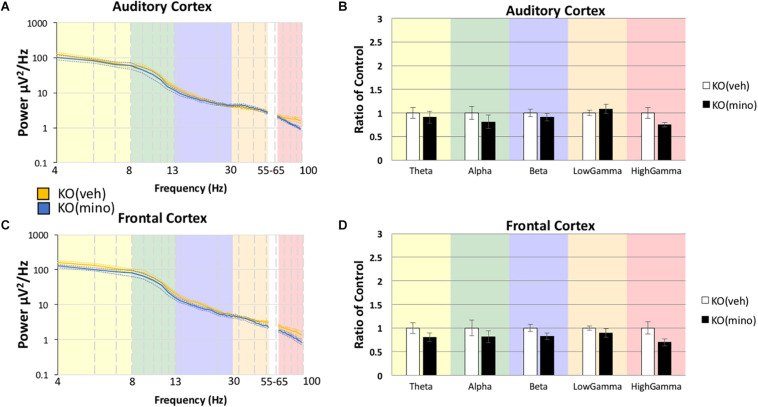
Minocycline effects on gamma power were no different from vehicle effects in the *Fmr1* KO mice. The 10-day minocycline effectiveness was compared against a 10-day vehicle treated group in *Fmr1* KO mice. Mean power density was calculated for both drug conditions using both auditory and frontal cortex electrodes **(A,C)**. No statistical significance was observed on this measure for KO mice **(B,D)**. Minocycline is not better or worse than a vehicle control on resting EEG power spectral density (12 minocycline KO and 11 vehicle KO; *p < 0.05, **p < 0.01, ***p < 0.001, ****p < 0.0001, ^#^*p* < 0.00001).

To determine if minocycline had a greater therapeutic effect than vehicle control we compared *Fmr1* KO mice treated with minocycline to *Fmr1* KO mice treated with vehicle (Figure 4B) in the AC (Box’s M, *p* = 0.458, Levene’s, all *p* > 0.05, Pillai’s Trace = 0.514, *p* = 0.05). No differences were observed in any frequency band, Theta *F*(1,18) = 0.167, *p* = 3.4385, η^2^ = 0.009, Alpha *F*(1,18) = 0.640, *p* = 2.1706, η^2^ = 0.034, Beta *F*(1,18) = 0.427, *p* = 2.6089, η^2^ = 0.023, Low Gamma *F*(1,18) = 0.452, *p* = 2.5500, η^2^ = 0.024, High Gamma *F*(1,18) = 3.185, *p* = 0.4560, η^2^ = 0.150. In the FC as well (Figure 4D, Box’s M, *p* = 0.089, Levene’s, all *p* > 0.05, Pillai’s Trace = 0.383, *p* = 0.1909), no differences were observed in any frequency band, Theta *F*(1,18) = 0.726, *p* = 2.0266, η^2^ = 0.039, Alpha *F*(1,18) = 0.292, *p* = 2.9790, η^2^ = 0.016, Beta *F*(1,18) = 1.812, *p* = 0.9747, η^2^ = 0.091, Low Gamma *F*(1,18) = 1.361, *p* = 1.2926, η^2^ = 0.070, High Gamma *F*(1,18) = 4.353, *p* = 0.2572, η^2^ = 0.195. These data indicate that both treatment types (vehicle and minocycline) reduce gamma band power in the *Fmr1* KO mice. Supplementary Figure S1 shows additional comparisons of resting EEG power to indicate that there were no effects of minocycline treatment compared to vehicle controls in either genotype, and that *Fmr1* KO mice have a significantly increased resting EEG power compared to WT, regardless of drug administration. Supplementary Figure S2 shows that if the drug effects were compared to pre-drug conditions, then minocycline does show a significant reduction of gamma power in the *Fmr1* KO mice but this effect is obscured when comparing only KO(veh) to KO(mino) as shown in Figure 4. One explanation is that the drug administration protocol itself and the direct effect of minocycline both have statistically trending effects given the sample sizes. By directly comparing pre-drug conditions to minocycline treatment combines both trending effects into a single significant observation. Considering the different comparisons possible, our overall interpretation is that minocycline does not have an additional impact on resting EEG than vehicle in the *Fmr1* KO mice in our study.

Delta power was analyzed separately because the data either did not meet assumptions of homogeneity of variance, normality, or both. Using non-parametric Mann-Whitney *U*-tests for each comparison on raw untransformed data, we observed that resting EEG delta power was different between the genotypes in the pre-drug condition in AC (*U* = 95, WT(pre) *n* = 21, KO(pre) *n* = 23, *p* = 0.0006) and FC (*U* = 90, WT(pre) *n* = 21, KO(pre) *n* = 23, *p* = 0.0004) (Supplementary Figure S3), and these differences were not altered by minocycline compared to WT vehicle controls: AC (*U* = 13, WT(veh) *n* = 12, KO(mino) *n* = 11, *p* = 0.0011) FC (*U* = 7, WT(veh) *n* = 12, KO(mino) *n* = 11, *p* = 0.0003) (Supplementary Figure S3). There was no difference between minocycline and vehicle treated KO mice: AC, U = 34, KO(veh) *n* = 10, KO(mino) *n* = 11, *p* = 0.1392; FC, *U* = 35, KO(veh) *n* = 10, KO(mino) *n* = 11, *p* = 0.1590 (Supplementary Figure S3). Thus, delta power is increased in *Fmr1* KO mice compared to WT, but minocycline did not correct this phenotype.

### Minocycline Improves ITPC in *Fmr1* KO Mouse Cortex Compared to Vehicle

**FIGURE 5:**
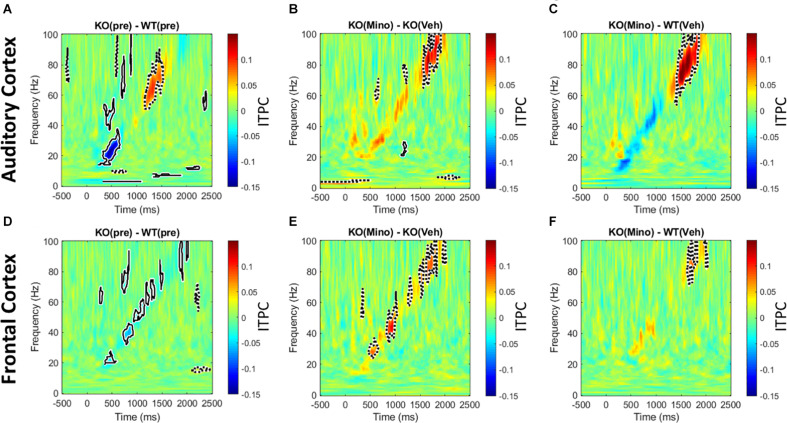
Minocycline reverses phase locking deficits tested with chirp stimuli. **(A–F)** Colored regions are group mean differences between different genotype and drug conditions. Black-outlined regions are significantly reduced (blue with solid black outline) or increased (red with dotted black outline) as a result of a statistical permutation approach. **(A, D)** In the AC, there is a decrease in ITPC at beta to low-gamma bands, and an increase in ITPC in the high gamma range in the *Fmr1* KO mice. In the FC, there is a decrease in ITPC between beta to high gamma frequencies. **(B, E)** Ten days of minocycline treatment increases ITPC in *Fmr1* KO mice compared to vehicle in the FC, but not in the AC, suggesting a differential response of the two regions to this treatment. **(C, F)** The normalizing effect of minocycline in *Fmr1* KO mice can be seen. In both the AC and FC, there is no difference between the minocycline treated KO and vehicle treated WT groups at beta/low-gamma bands.

The genotype differences in chirp response ITPC in the pre-drug condition (Figures 5A,D) were mostly consistent with our previous report (Lovelace et al., 2018). In the AC, ITPC was lower in the beta and low-gamma bands in the KO mice. In the FC, ITPC was lower from beta to high-gamma frequencies in the KO mice. One difference between the present data and our published work was the increase in ITPC in *Fmr1* KO mice at high gamma frequencies (∼60–80 Hz) in the AC. Minocycline treatment of *Fmr1* KO mice significantly increased ITPC (Figures 5B,E) compared to vehicle treated *Fmr1* KO mice across beta to gamma bands (∼20–100 Hz) in the FC, and in the high gamma band (∼80–100 Hz) in the AC. A comparison of *Fmr1* KO mice treated with minocycline to vehicle treated WT mice (Figures 5C,F) shows no difference in ITPC in the AC between ∼20–40 Hz (where prominent reductions were observed in pre-drug condition). But since minocycline did not significantly increase ITPC over vehicle in this region (∼20–40 Hz) (Figure 5B), this normalization of the phenotype does not appear to be better than vehicle treatment. However, in the FC, minocycline treatment significantly increased ITPC across beta-gamma frequencies compared to vehicle treatment in *Fmr1* KO mice (Figure 5E) and no significant differences in ITPC were observed across beta-low gamma frequencies when comparing minocycline treated *Fmr1* KO mice to vehicle treated WT controls (Figure 5F). Supplementary Figure S4 (A–F) shows a different method of plotting the same data to better illustrate the effects of minocycline on chirp ITPC only for the frequencies present in the chirp at any given time (i.e., along the diagonal of time-frequency response in Figure 5). Plots in S4 show the mean and standard error of ITPC of chirp response across the drug conditions, and allow the variability within groups to be visualized. These data indicate that minocycline was more effective in increasing phase coherence to time-varying stimuli when compared to vehicle treatment.

### Minocycline Improves ASSR ITPC in *Fmr1* KO Mouse Cortex Compared to Vehicle

**FIGURE 6:**
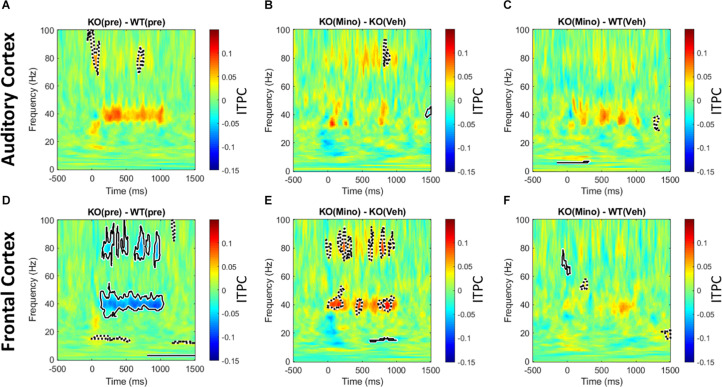
Minocycline reverses phase locking deficits in the 40 Hz ASSR in the FC. An auditory steady state response is the response to a series of 40 Hz train of clicks (1 s duration). The analysis and interpretation are the same as in Figure 5. Comparing genotype differences in ITPC before drug treatment reveals a robust reduction in the *Fmr1* KO mice compared to WT in the FC **(D)**, but not in the AC **(A)**. After treating KO mice with minocycline for 10 days, ITPC values in the FC increased significantly compared to vehicle-treated controls in matching time x frequency regions **(E)**, with no effect in the AC **(B)**. Direct comparison of ASSR in *Fmr1* KO mice treated with minocycline to vehicle-treated WT mice show virtually no difference, suggesting a rescue of the phenotype in the FC **(F)**, and again no effect found in the AC **(C)**.

The 40 Hz auditory steady state response (ASSR) was compared between the *Fmr1* KO and WT mice in the pre-drug condition. There is a significant reduction of ITPC in the FC of the *Fmr1* KO mice (Figure 6D), but not in the AC (Figure 6A). After treating *Fmr1* KO mice with minocycline for 10 days, ITPC values in the FC were significantly increased over vehicle treated KO mice (Figure 6E). Finally, direct comparison of the *Fmr1* KO mice treated with minocycline to WT vehicle treated group show no statistical difference (Figure 6F). To complement the cluster analysis plots in Figure 6 and Supplementary Figure S4 (G-L) show the group mean and standard error of ITPC in the ASSR using the same data from Figure 6. These plots allow the variability within groups to be visualized. Together, these data reveal a novel genotype and cortical region specific ITPC deficit in the 40 Hz ASSR, and that minocycline can reverse these deficits compared to vehicle treatment.

### Minocycline Reduces Sound-Induced Baseline Corrected Gamma Power in *Fmr1* KO Mice Compared to Vehicle

**FIGURE 7:**
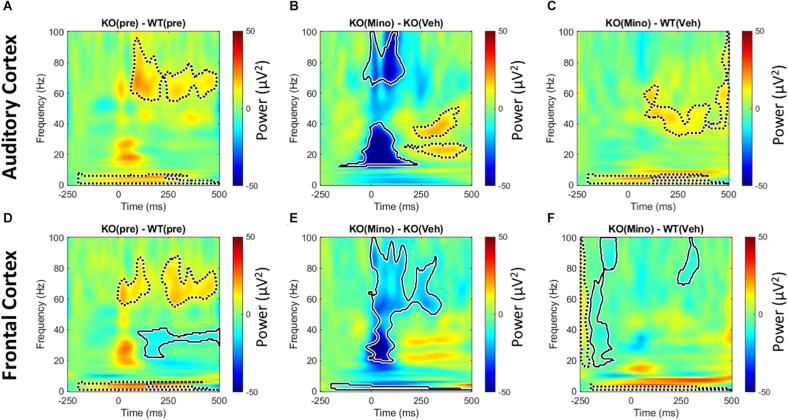
Minocycline reduces sound-induced gamma power in *Fmr1* KO mice. Trains of 100 ms duration broad band noise were used to generate event related potentials (200 repetitions) for time × frequency analyses. After correcting for baseline (–250 ms through –150 ms) single trial power was averaged over all trials. This allows for the analysis of power that is normalized to individual animal baselines. In the pre-drug condition, *Fmr1* KO mice showed increased on-going power after the auditory stimulus in the delta and high gamma range in both AC and FC **(A,D)**. In addition, the FC showed a significant reduction in power the beta to low gamma frequency range **(D)**. Minocycline treatment significantly reduced onset power in *Fmr1* KO animals over vehicle treatment in both FC and AC **(B,E)**, but also increased power in the AC in two distinct bands at ∼20 and ∼40 Hz that occurred well after sound onset. Finally, comparing minocycline treated *Fmr1* KO mice to vehicle-treated WT group, reveals a shift in power in AC from high gamma in **(A)**, to low gamma in **(C)**. Additionally in the FC, ongoing gamma power and modulation of beta – low gamma power in **(D)** are significantly reduced **(F)**.

Trains of 100 ms duration broad band noise bursts were presented to mice (200 repetitions). After correcting for baseline (−250 ms through −150 ms) single trial power was averaged over all trials. This allows for the analysis of sound-induced power that is normalized to the baseline for each animal. Consistent with previous report (Wen et al., 2019), we found a genotype difference with the *Fmr1* KO mice showing on-going increase in power in the delta and 60–80 Hz gamma band in the 100–400 ms time window after stimulus onset (Figures 7A,D). This was seen in both regions, with the FC also showing an ongoing decrease in beta/low gamma band power. Minocycline treatment drastically reduced onset power in *Fmr1* KO mouse responses over vehicle treatment in both FC and AC (Figures 7B,E). There was also a reduction in the ongoing 60–80 Hz gamma power. In the AC, however, there was increased ongoing power in 2 distinct lower-frequency bands at ∼20 Hz and ∼40 Hz. Finally, comparison of the minocycline-treated *Fmr1* KO mice to WT vehicle treated group reveals a shift in power in AC from high gamma to low gamma bands. Additionally, in the FC, ongoing gamma power and reduced beta – low gamma power were normalized (Figures 7C,F). We interpret these data to indicate a beneficial effect of minocycline in both AC and FC by reducing stimulus-evoked onset power and longer latency induced power in the high gamma frequencies.

### Additional Analysis of Movement During EEG Recordings

While the FFT analysis of resting EEG used movement as a covariate, it is possible that movement itself was affected across recording sessions and/or within sessions due to auditory stimulus presentation. Supplementary Figure S5A shows the percentage of time mice moved during resting EEG across genotypes and treatment. First, we determined that KO mice moved less than WT during pre-drug treatment using a *t*-test, *t*(42) = 2.741, *p* = 0.0090. Then to determine drug effects, we used time spent moving during pre-drug treatment as a co-variate in a two-way ANCOVA on Genotype X Drug, this controls for the original movement differences found in the pre drug condition. There was no effect of Genotype *F*(1,37) = 1.187, *p* = 0.2829, Drug *F*(1,37) = 0.676, *p* = 0.4163, or Genotype X Drug interaction *F*(1,37) = 0.013, *p* = 0.9105. Therefore, the EEG changes due to minocycline are unlikely to be due to the drug’s effect on movement or due to changes in movement across recording conditions.

To determine if exposure to sound altered movement of the animals, we recorded the % time mice spent moving in 5 s windows of silence between presentations of 100 trains of sounds over a period of sound evoked recordings (∼52 min). KO mice also move less than WT mice during sound presentation, *t*(42) = 5.051, *p* = 0.000009, two-way ANCOVA on Genotype X Drug using movement during pre as a co-variate shows no effect of Genotype *F*(1,37) = 0.263, *p* = 0.6114 or Drug *F*(1,37) = 3.319, *p* = 0.0765, but there was a significant Genotype × Drug interaction *F*(1,37) = 4.8790, *p* = 0.0335. Further analysis of simple effects shows that minocycline had a trending effect on reducing WT movement during sound presentation *F*(1,18) = 4.346, *p* = 0.0516, but had no effect on KO mice *F*(1, 18) = 0.016, *p* = 0.8993. Therefore, the effects of minocycline during sound presentation in the KO mice is unlikely to be due to altered movements by the sound. To identify if auditory stimulation within a recording session affected movement, we analyzed movement in the silent intervals during acoustic stimulation for the first 10 and the last 10 trains of sounds (Supplementary Figures S5C,D). Yet again, the data does not show any effects of minocycline on movement within a recording session in the Fmr1 KO mice.

## Discussion

We studied the effects of 10-day minocycline treatment in adult mice on resting EEG power, phase locking in the response to auditory chirp stimuli and 40 Hz ASSR, and on the induced power in response to noise burst stimuli. These responses provide insight into abnormalities in background cortical activity, temporal fidelity in evoked synchronization and sound-induced power, respectively, in *Fmr1* KO mice, and serve to identify neural correlates of abnormal auditory processing in FXS. We replicated many of the previously reported phenotypes (Lovelace et al., 2018) in this new cohort of *Fmr1* KO mice. This includes increased gamma band resting EEG power, reduced ITPC to chirp and increased ongoing induced gamma power (Lovelace et al., 2018; Wen et al., 2019). We also report a novel 40 Hz ASSR deficit with a reduction in ITPC in the FC, but not AC, of *Fmr1* KO mice compared to WT mice. The major finding in terms of minocycline treatment is that sound-evoked, but not resting, EEG phenotypes in adult *Fmr1* KO mice are more sensitive to minocycline treatment than vehicle. This includes improved fidelity of evoked synchronization with chirps and ASSR, and reduced induced power.

Minocycline does not change resting EEG power distribution in a manner that is different from vehicle treatment in the *Fmr1* KO mice. Even though the drug reduced resting EEG gamma power in the *Fmr1* KO mice to vehicle treated WT levels, vehicle treatment in the KO mice had the same effect. This effect was seen in both AC and FC. This suggests that other factors, such as the treatment protocol (including stress and arousal of daily handling and oral gavage protocols), can influence resting EEG responses in the *Fmr1* KO mice. Even though the *Fmr1* mice were given 15 min of habituation in the recording chamber after the electrodes were connected, it is possible that their baseline arousal state is higher when resting EEGs were recorded compared to WT mice. Resting EEG rhythms can be altered by neuromodulators that promote arousal such as acetylcholine and influence drug x genotype interactions (Babiloni et al., 2013). Sound evoked oscillations may have a larger magnitude, compared to high frequency resting EEG components, to reveal sensitivity to minocycline compared to vehicle.

In terms of evoked responses, a significant effect of minocycline treatment in *Fmr1* KO mice compared to vehicle treatment was the improvement in ITPC for chirp response. Ethridge et al. showed that ITPC is reduced in humans with FXS. Likewise, Lovelace et al. (2018) showed a reduction in ITPC to the chirp in the *Fmr1* KO mice. ITPC measures the ability of evoked oscillations to phase lock with fidelity from one trial to the next. Reduced ITPC indicates that the neural generators show higher jitter in their phase locking to repeated temporally modulated stimuli. In both humans with FXS and *Fmr1* KO mice, the reduction was robust in the 30-50 Hz range suggesting a gamma band deficit.

Gamma band ITPC deficit in chirp response is further substantiated by the observed 40 Hz ASSR ITPC deficits in *Fmr1* KO mice. ASSR may be a useful translational biomarker as robust differences are seen in neurodevelopmental disorders including schizophrenia and autism. In schizophrenia, patients show reduced phase locking to 40 Hz stimulation (Light et al., 2006). Studies in children with autism, and their unaffected first degree relatives, showed a reduced 40 Hz ASSR power and phase locking compared to age-matched controls (Wilson et al., 2007; Rojas et al., 2008). Consistent with this, we observed a reduction in ITPC in the 40 Hz ASSR in *Fmr1* KO mice. Whether children with FXS also show abnormal ASSR has not been tested, but the mouse results suggest that this may be a potentially useful avenue for clinical applications in humans with FXS.

The ITPC deficits for both the chirp response and ASSR are reversed by minocycline treatment. In both cases, minocycline-treated KO mice showed improved responses compared to vehicle-treated KO group suggesting a true drug effect. The gamma band deficits in ITPC suggests that minocycline may act by affecting PV cell function in *Fmr1* KO mice. There is converging evidence for dysfunction of PV neurons in *Fmr1* KO mice, and this has been seen across auditory, somatosensory and visual cortices (Selby et al., 2007; Nomura et al., 2017; Goel et al., 2018; Wen et al., 2018). Abnormal PV cell function may be related to reduced integrity of perineuronal nets (PNN). PNNs are specialized assemblies of extracellular matrix components that preferentially form around PV neurons (Brewton et al., 2016; Lensjø et al., 2017; Nguyen et al., 2017; Ueno et al., 2018; Wen et al., 2018). Loss of PNNs around PV cells is predicted to reduce PV expression, as well as excitability of these interneurons (Balmer, 2016). MMP-9 cleaves components of extracellular matrix and increased activity of MMP-9 in FXS may lead to reduced PNN expression and abnormal function of PV cells. Indeed, genetically reducing MMP-9 in *Fmr1* KO mice normalizes PV/PNN development and electrophysiological responses in the auditory cortex of *Fmr1* KO mice. One known effect of minocycline is the inhibition of MMP-9 (Dziembowska et al., 2013). However, minocycline has other effects in the nervous system including modulation of cell apoptosis, inflammation and microglial clearance (Dean et al., 2012). EEG data obtained using SB-3CT, a specific inhibitor of MMP-9, show reversal of multiple EEG phenotypes (Pirbhoy et al., 2020). This adds strength to the notion that reduction of neural correlates of auditory hypersensitivity by minocycline involves MMP-9 inhibition. Therefore, the reduction of MMP-9 and normalization of PV neuron function may underlie beneficial effects of minocycline treatment, which manifests as normalized gamma band synchrony in both ASSR and chirp responses.

Another major effect of minocycline treatment was reduction in stimulus evoked and induced ERP power. With time/frequency analysis of auditory ERPs, two types of gamma oscillations can be distinguished. The short latency (<100 ms) response is tied to stimulus onset, while the longer latency (150–400 ms) gamma oscillation contains induced power that is not stimulus locked (Franowicz and Barth, 1995; Crone et al., 2001; Brosch et al., 2002; Jeschke et al., 2008). Using a cortical slice preparation, Metherate and Cruikshank (1999) showed that thalamic stimulation caused an early glutamatergic potential in the input layer of auditory cortex. This was followed by long-latency gamma-band fluctuations driven by polysynaptic activity (akin to induced power). This long-latency gamma band activity was dominant in the middle and superficial layers and propagated intracortically. This may facilitate formation of transient neural assemblies by synchronizing activity across rapidly changing pools of neurons. The gamma fluctuations consisted of both fast depolarization and GABAergic inhibition. Given the broader tuning curves of cortical neurons in the *Fmr1* KO mice, and increased synchrony across neurons (Goswami et al., 2019), the transient neural assemblies formed in the 150–400 msec window after stimulus onset may incorporate the activity of a larger number of neurons with overlapping tuning. This would produce the observed increase in induced gamma power.

Minocycline treatment in KO mice reduced both sound-evoked onset and ongoing induced power when compared to vehicle control. One interpretation is that by reducing the magnitude of the onset response, the intracortical spread of polysynaptic activity and synchronized neural pool recruitment is reduced. Additionally, there were no phase-locking differences in sound-evoked ERPs between WT and KO pre-drug, indicating that changes in sound-evoked power were primarily driven by increased magnitudes of individual responses, and not inter-trial synchrony. However, therapeutic reduction in sound-evoked power by minocycline was associated with a reduction in ITPC, which indicates that even though there was no difference in ITPC pre-drug, the mechanism of minocycline action seems to be through desynchronization of neural populations, which may contribute to a smaller evoked response, and subsequently preventing the network from entering a persistently active state.

In general, FC appeared to be more malleable in response to minocycline than AC. It is possible that AC requires earlier time points of treatment compared to the FC. Indeed, Pirbhoy et al. (2020) showed that the treatment of young (P21–22) mice with SB-3CT, a selective MMP-9 inhibitor, increases ITPC of chirp responses around 40 Hz, and increases the density of PV and PNN expressing cells in the AC. Regional differences may also arise because the neural generators that give rise to gamma oscillations in the AC are different from the FC. Sound-evoked oscillations recorded from the AC are likely driven by a mixture of primary cortical neural generators and from multiple subcortical structures (Bidelman, 2018), while FC gamma oscillations are less likely to be directly influenced by auditory subcortical sites. However, FC gamma oscillations can be strongly modulated by projection PV cells in the basal forebrain, in a state-dependent manner associated with arousal and attention (Kim et al., 2015). Evidence of such a possibility can be seen by comparing induced power to sound bursts in *Fmr1* KO mice treated with minocycline in this study, to transgenic mice which have a specific cortical KO of *Fmr1* (Lovelace et al., 2019). The correlation between these 2 groups on this measure is striking, including increased power in distinct bands at ∼20 and ∼40 Hz post stimulation, in addition to a reduction in ITPC during sound onset in the AC. One interpretation is that minocycline could have its therapeutic effect at treating local cortical circuits or basal forebrain structures, and less of an effect on subcortical auditory pathways. Future studies will more systematically examine the role of treatment age on regional brain effects, and the longevity of the beneficial effects.

### Clinical Relevance

Sensory hypersensitivity is a debilitating and consistent symptom of FXS, and auditory hypersensitivity is a prominent manifestation noted in clinical and parent reports. Early childhood sensory processing abnormalities may lead to increased anxiety and delayed language development. In the mouse model of FXS, we have identified four neural correlates of auditory hypersensitivity: increased single neuron responses to sounds (Rotschafer and Razak, 2013; Wen et al., 2019; Nguyen et al., 2020), more neurons activated by a single sound (Rotschafer and Razak, 2013; Nguyen et al., 2020), reduced habituation of cortical responses to repeated sounds (Lovelace et al., 2016) and abnormal synchrony of sound driven responses (Lovelace et al., 2018, 2019; Wen et al., 2019). Either genetic reduction of MMP-9 (Lovelace et al., 2016; Wen et al., 2018) or reduction of MMP-9 with a specific inhibitor (Pirbhoy et al., 2020) or with the FDA-approved minocycline normalized these abnormal responses (present study). Taken together with studies that observed improved outcomes in humans with FXS (Paribello et al., 2010; Leigh et al., 2013; Schneider et al., 2013) and the drosophila model of FXS (Siller and Broadie, 2011), these data point to the strong potential of MMP inhibition as a therapeutic avenue to reduce sensory symptoms in humans with FXS.

An urgent need in neurodevelopmental disorders research and drug development is the identification of objective, robust, and translation-relevant biomarkers (Sahin et al., 2018). EEG phenotypes may serve this need, given the consistency in both type and direction of abnormalities in *Fmr1* KO mice and humans with FXS. Moreover, EEG phenotypes recorded in humans with FXS are correlated with abnormal sensory sensitivity reports and deficits in social communication as measured with the social and communication questionnaires. These correlations suggest clinical significance of EEG phenotypes in humans and point to the utility of EEG measures for both stratification and for measuring drug efficacy in clinical trials. An important aspect of biomarker development is that the specificity and sensitivity of drug effects (over placebo effects) can be quantified in both mice and humans. Our data from the *Fmr1* KO mouse suggest that minocycline has spectral band- and EEG response-specific effects. Future studies in humans with FXS should examine the effects of minocycline on a range of EEG measures in humans. So far, published human data (Schneider et al., 2013) have only examined sound evoked habituation, and showed that minocycline, compared to placebo, improved ERP N1 component habituation to repeated sounds in patients with FXS. Whether phase-locking and resting EEGs are also improved in humans remains to be tested.

Minocycline has been tested as a potential therapeutic in a number of neurodevelopmental and neurodegenerative conditions including Angelman syndrome (Grieco et al., 2014), schizophrenia (Chaudhry et al., 2012), depression (Miyaoka et al., 2012), Parkinson’s disease (Thomas and Le, 2005) and Alzheimer’s disease (Choi et al., 2007). EEG recordings have been used to obtain physiological markers in these conditions (Sponheim et al., 1994, 2000; Narayanan et al., 2014). Some of the phenotypes may be used as outcome measures for candidate drug testing. Our results suggest that an evaluation of how minocycline affects a broad range of EEG phenotypes in preclinical models is required before designing outcome measures in humans. Different EEG responses and cortical regions are differentially susceptible to the specific treatment protocol used. This is an important consideration moving forward with the use of EEGs in clinical trials.

## Data Availability Statement

The raw data supporting the conclusions of this article will be made available by the authors, without undue reservation.

## Ethics Statement

The animal study was reviewed and approved by IACUC, UCR.

## Author Contributions

JL, DB, IE, and KR designed the study and worked together to write the manuscript. JL performed the experiments and analyzed the data. All authors contributed to the article and approved the submitted version.

## Conflict of Interest

The authors declare that the research was conducted in the absence of any commercial or financial relationships that could be construed as a potential conflict of interest.
