# Supplementary material for: Minocycline Treatment Reverses Sound Evoked EEG Abnormalities in a Mouse Model of Fragile X Syndrome
Source: Front Neurosci. 2020 Aug 4;14:771. doi: 10.3389/fnins.2020.00771 (PMC7417521; doi:10.3389/fnins.2020.00771)
Supplement: Supplementary file 1 [file Data_Sheet_1.docx]

**
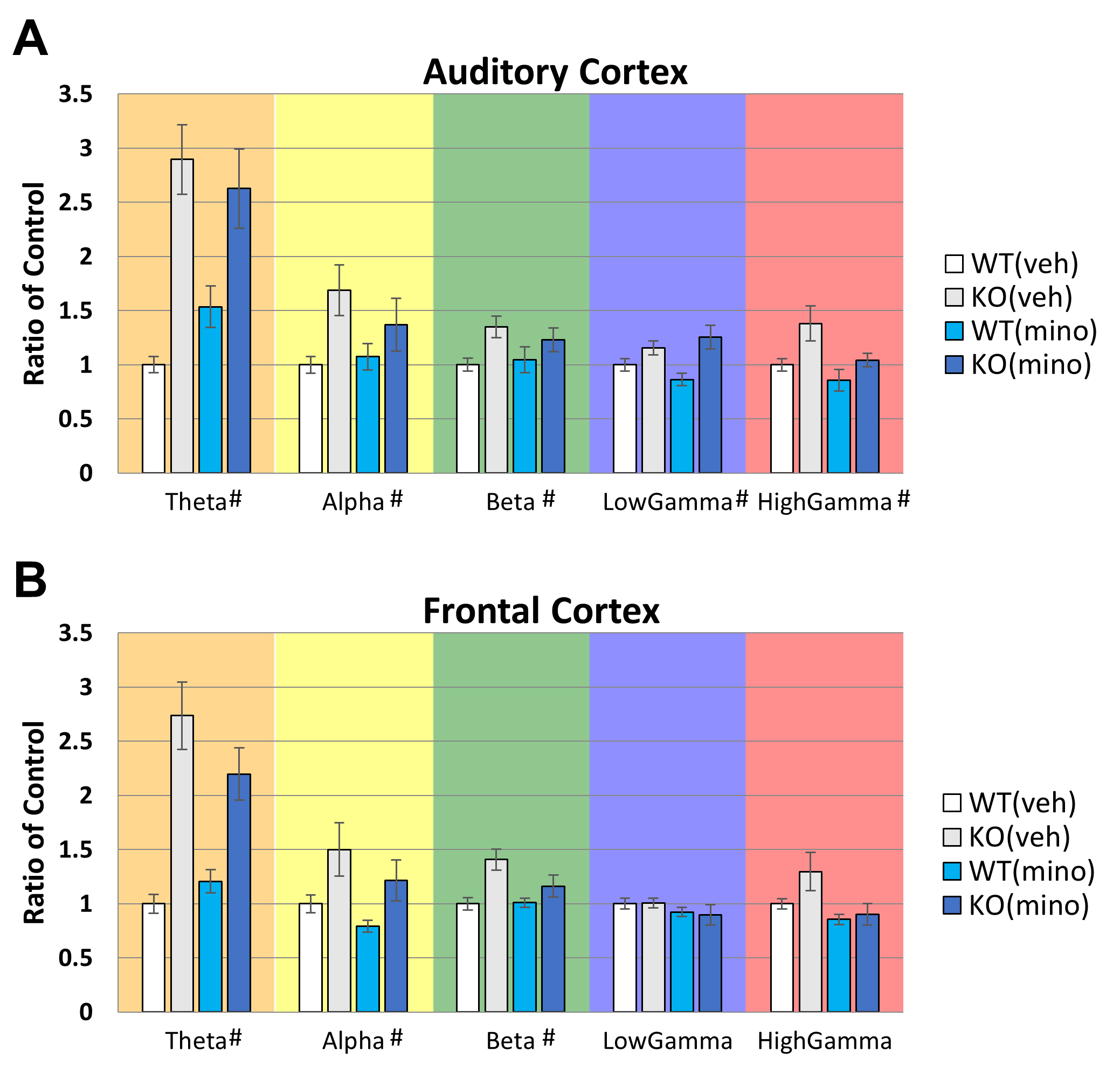
**

**Supplemental Figure 1. Post treatment only reveals effects of genotype.**

To clarify drug and genotype effects, a two-way MANCOVA analysis of Genotype x Drug was run including movement as a covariate. In the auditory cortex (A) we confirmed assumptions of equality of covariance using Box’s M, p = 0.060, as well as Levene’s test of equality of error variances. No differences between the three drug conditions were observed in error variance (all p > 0.05). Since assumptions were not violated, we report an effect of Genotype (Pillai’s Trace = 17.301, p = 2.1497 x 10^-8^), Drug (Pillai’s Trace = 2.872, p = 0.0292), and interaction (Pillai’s Trace = 2.609, p = 0.0428), across all 5 of the combined frequency variables. However, after post hoc analysis of each frequency band, only main effects of genotype remained: Theta, F(1,37) = 36.992, p = 2.4306 x 10^-6^, ƞ^2^ = 0.500, Alpha, F(1,37) = 8.069, p = 0.0364, ƞ^2^ = 0.179, Beta, F(1,37) = 9.004, p = 0.0240, ƞ^2^ = 0.196, Low Gamma, F(1,37) = 16.195, p = 0.0014, ƞ^2^ = 0.304, and High Gamma, F(1,37) = 10.699, p = 0.0116, ƞ^2^ = 0.224. No drug or interaction effects were observed for any individual frequency band (all p > 0.05 after Bonferroni correction). In the frontal cortex (B) we report an effect of Genotype (Pillai’s Trace = 10.625, p = 3.8379 x 10^-6^), and interaction (Pillai’s Trace = 2.666, p = 0.0394), but no main effect of Drug (Pillai’s Trace = 1.661, p = 0.172), across all 5 of the combined frequency variables. However, after post hoc analysis of each frequency band, only main effects of genotype remained in: Theta, F(1,37) = 45.388, p = 3.1838 x 10^-7^, ƞ^2^ = 0.551, Alpha, F(1,37) = 9.677, p = 0.0179, ƞ^2^ = 0.207, and Beta, F(1,37) = 10.722, p = 0.0115, ƞ^2^ = 0.225. We interpret these results to indicate that there are no effects of minocycline treatment compared to vehicle controls, and that KO mice have a significant increased power compared to WT, regardless of drug administration. P-values were corrected for multiple comparisons using Bonferroni procedures, and each brain region was run separately. #main effect of genotype.


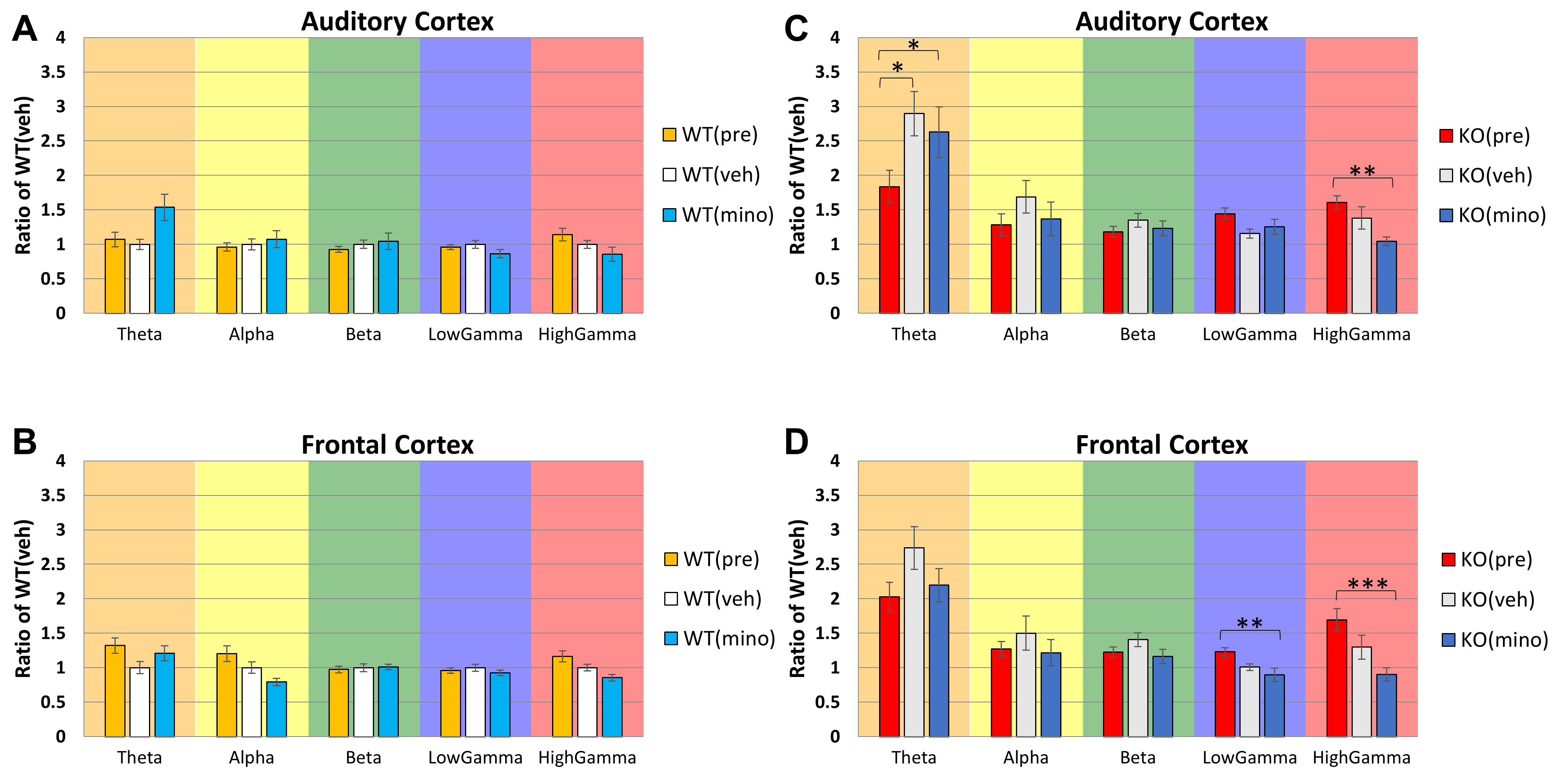


**Supplemental Figure 2. Minocycline reduces gamma band power only in *Fmr1* KO mice when compared to pre-drug treatment.**

To determine if the drug administration protocol had any effects on WT mice MANCOVA analysis was run on WT mice comparing pre-treatment and both drug conditions (three levels: Pre, Veh, and Mino). In the auditory cortex of WT mice (A) we confirmed assumptions of equality of covariance using Box’s M, p = 0.104, as well as Levene’s test of equality of error variances. No differences between the three drug conditions were observed in error variance (all p > 0.05). Since assumptions were not violated, we report no effect of drug administration (Pillai’s Trace = 0.381, p = 0.1118) across all 5 of the combined frequency variables, which include movement as a covariate, since no effect was seen at the multivariate level, no further analysis was conducted. In the frontal cortex of WT mice (B) we confirmed assumptions of equality of covariance using Box’s M, p = 0.326, as well as Levene’s test of equality of error variances. No differences between the three drug conditions were observed in error variance (all p > 0.05). Since assumptions were not violated, we report an effect of drug administration (Pillai’s Trace = 0.711, p = 0.0003) across all 5 of the combined frequency variables, which include movement as a covariate. However, after Bonferroni correction for multiple comparisons, no individual frequency band had an adjusted p-value that was <0.05. To determine if the drug administration protocol had an effect on KO mice MANCOVA analysis was run on only KO mice comparing pre-treatment and both drug conditions. In the auditory cortex of KO mice (C) we confirmed assumptions of equality of covariance using Box’s M, p = 0.763, as well as Levene’s test of equality of error variances. No differences between the three drug conditions were observed in error variance (all p > 0.05) Since assumptions were not violated, we report an effect of drug administration (Pillai’s Trace = 5.506, p = 4.4753 x 10^-6^) across all 5 of the combined frequency variables, which include movement as a covariate. The only frequency bands that showed post-hoc effects were Theta, F(2,40) = 5.399, p = 0.0420, ƞ^2^ = 0.213, and High Gamma F(2,40) = 7.625, p = 0.0078, ƞ^2^ = 0.276. Further pairwise post hoc comparisons in the theta band revealed increases in power after both vehicle treatment (p = 0.026) and minocycline (p=0.047). The high gamma band revealed only a difference in minocycline (p = 0.0012) and not vehicle (p = 0.2877), when compared to pre-drug. In the frontal cortex of KO mice (D) we also report an effect of drug administration (Pillai’s Trace = 5.506, p = 7.5956 x 10^-5^). The only frequency bands that showed post-hoc effects were Low Gamma, F(2,40) = 7.320, p = 0.0098, ƞ^2^ = 0.268, and High Gamma F(2,40) = 9.989, p = 0.0015, ƞ^2^ = 0.333. Further pairwise post hoc comparisons in the low gamma band revealed decreases in power after minocycline (p = 0.0022), but not vehicle (p = 0.1061). Similarly, the high gamma band revealed a difference in minocycline (p = 0.0002) and not vehicle (p = 0.1528). Taken together, these results indicate that minocycline is more effective than vehicle treatment in reducing gamma power when compared to pre-drug conditions, but this effect is obscured when comparing only KO(veh) to KO(mino) (Figure 4). P-values were corrected for multiple comparisons using Bonferroni procedures, and each brain region was run separately. *p<0.05, **p<0.01, ***p<0.001.


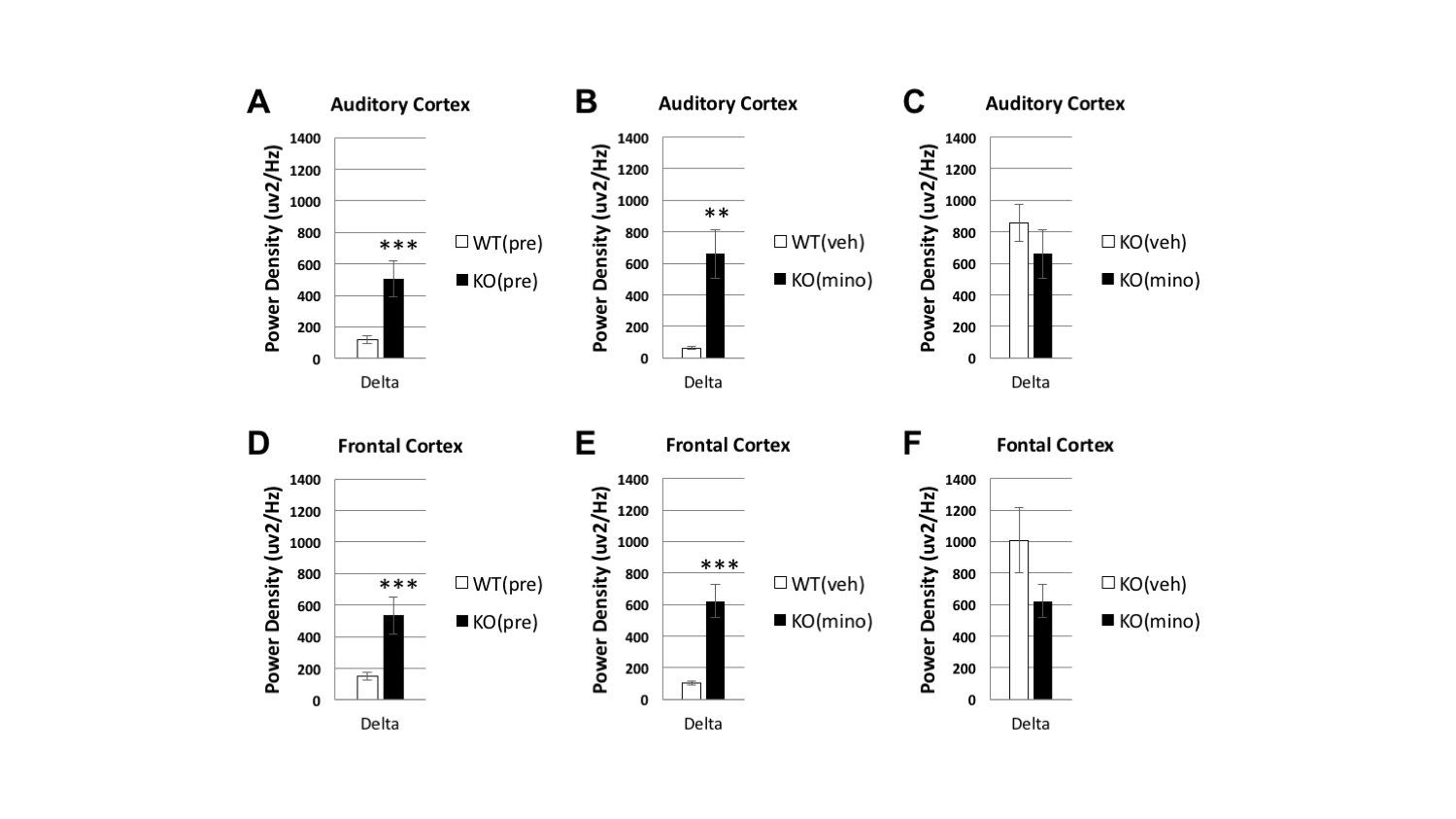


**Supplemental Figure 3. *Fmr1* KO mice have increased resting EEG delta power compared to WT with no effect of drug treatment.**

Delta is analyzed here with the same group comparisons and order as in Figures 2-4. Statistics on Delta was run separately because this frequency band did not meet assumptions of MANCOVA analysis. Here non-parametric Mann-Whitney U tests are run for each comparison. KO mice had large significant increases in raw delta power in both brain regions pre drug (A&F), and it remained high after minocycline treatment (B&E), however no differences were found between KO animal groups treated with minocycline and vehicle controls (C&F). *p<0.05, **p<0.01, ***p<0.001, ****p<0.0001, #p<0.00001.


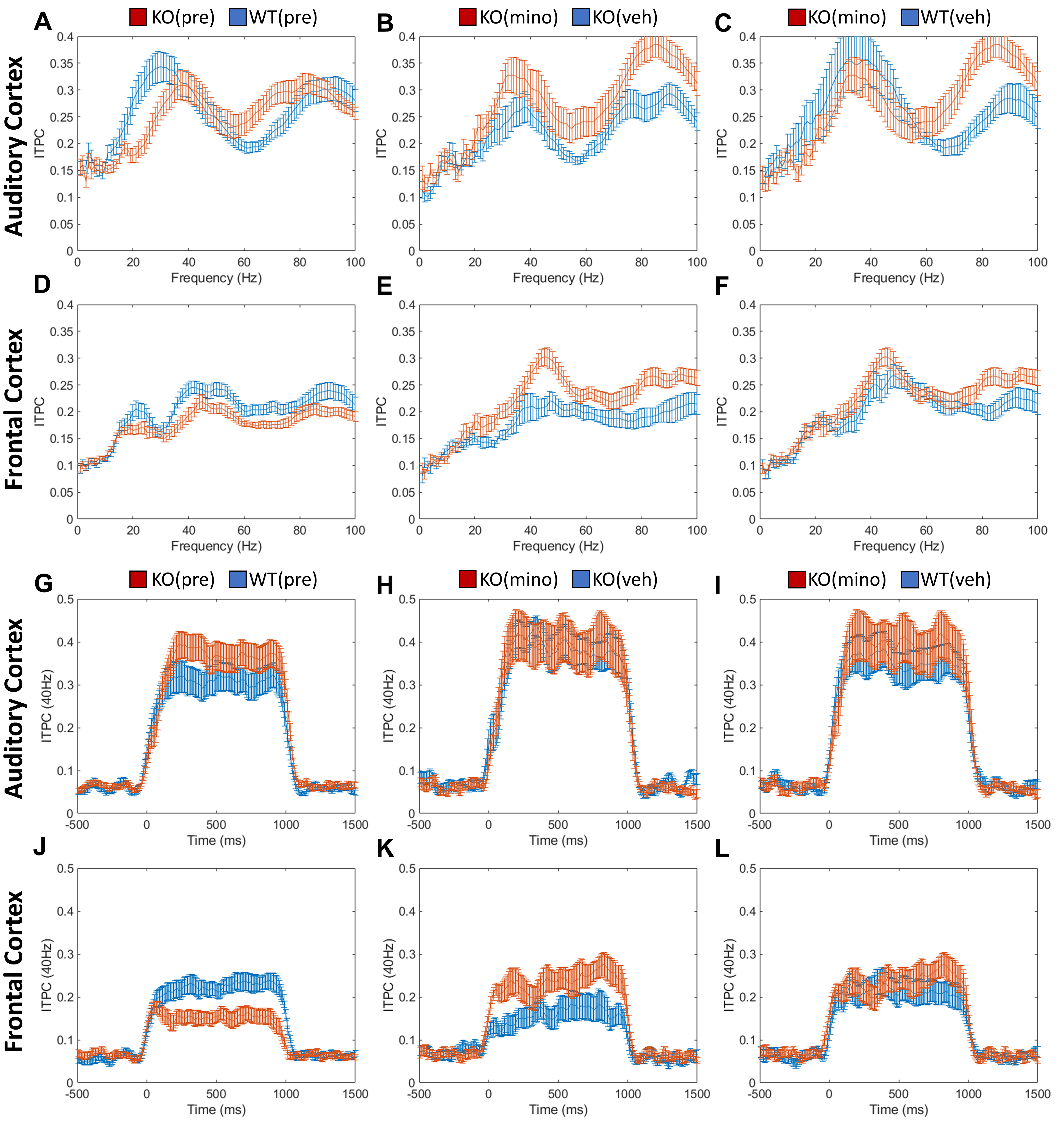


**Supplemental Figure 4. Minocycline reverses phase locking deficits in KO mice on auditory chirp stimuli and 40Hz ASSR**

(A-F) The data that has been shown in Figure 5 on the “up-chirp” is shown here, except ITPC is graphed only along the diagonal line in the Time X Frequency domain which is aligned with the up-chirp stimulus. (origin starting from 0ms ,0Hz and extending linearly up to 2000ms, 100Hz). Letters in the figure correspond to the matching letters in Figure 5. Graphing the data in this manner allows for the variability in the data to be visualized and assessed for effect sizes. Each data point is the group mean ITPC at each frequency along the diagonal with error bars indicating SEM. (G-L) The data that has been shown in Figure 6 on the “40Hz ASSR” is shown here, except ITPC is graphed only along the 40Hz frequency band, with time still being the same on the x-axis. Graphing the data in this manner allows for the variability in the data to be visualized and assessed for effect sizes. Each data point is the group mean ITPC at each time point with error bars indicating SEM.


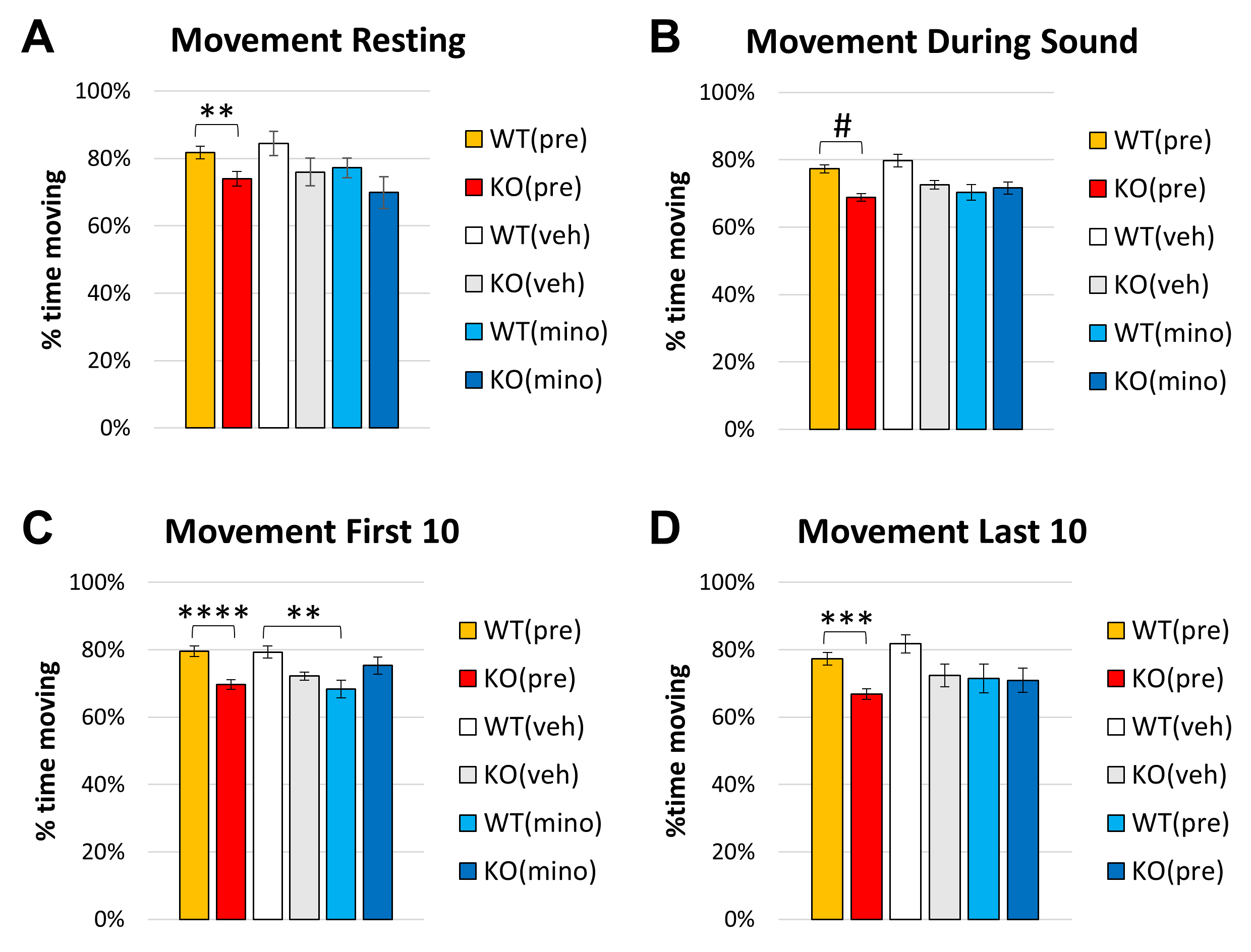


**Supplemental Figure 5. Analysis of movement across sessions and due to auditory stimulation**

Movement during the first 5 minutes of resting EEG was simultaneously recorded. A piezoelectric transducer was placed underneath the arena to detect movement. This is also the movement variable that was used as a covariate for statistical analysis of FFT during rest. (A) Here we show the percent of time mice spent moving during the initial resting period (after 15-20mins of habituation). First, we determined that KO mice moved less than WT during pre-drug treatment using a t-test, t(42) = 2.741, p = 0.0090. Then to determine drug effects, we used time spent moving during pre-drug treatment as a co-variate in a two-way ANCOVA on Genotype X Drug, this controls for the original movement differences found in the pre drug condition. There was no effect of Genotype F(1,37) = 1.187, p = 0.2829, Drug F(1,37) = 0.676, p = 0.4163, or Genotype X Drug interaction F(1,37) = 0.013, p = 0.9105. (B) To determine if exposure to sound altered movement of the animals, we recorded the % time mice spent moving in 5 second windows of silence between presentations of 100 trains of sounds over a period of ≈52min. KO mice also move less than WT mice during sound presentation, t(42) = 5.051, p = 0.000009, two-way ANCOVA on Genotype X Drug using movement during pre as a co-variate shows no effect of Genotype F(1,37) = 0.263, p = 0.6114 or Drug F(1,37) = 3.319, p = 0.0765, but there was a significant Genotype X Drug interaction F(1,37) = 4.8790, p = 0.0335. Further analysis of simple effects shows that minocycline had a trending effect on reducing WT movement during sound presentation F(1,18) = 4.346, p = 0.0516, but had no effect on KO mice F(1, 18) = 0.016, p = 0.8993. (C) In order to observe movement effects within recording sessions, we measured % movement during the first 10 sound trains (or for the first ≈5.2min of sound exposure). Again KO mice moved less than WT in pre drug conditions, t(42) = 4.611, p = 0.000037. Using the same two-way ANCOVA approach as above we report a Genotype X Drug interaction F(1,37) = 12.273, p = 0.0012. Simple effects again show that minocycline reduces WT movement F(1,18) = 10.244, p = 0.0050, but had no effect on KO movement F(1,18) = 0.034, p = 0.8555. (D) We then measured percentage movement during the last 10 sound trains (or after ≈46.5min of sound exposure). KO mice moved less than WT, t(1,42) = 4.1877, p = 0.0001. Two-way ANCOVA during the last 10 trains showed no main effect of Genotype F(1,37) = 0.5529, p = 0.4618, or Drug F(1,37) = 1.7763, p = 0.1908, and no interaction F(1,37) = 1.5523, p = 0.2206. Finally, we compared data from (C) and (D) using repeated measures ANOVA, Time X Genotype for the pre drug conditions, and then Time X Genotype X Drug for post drug conditions. Time in this case is referring to movement during the start of sound presentation, and movement during the end of sound presentation. No main effects of Time, Genotype, Drug, or any interactions were observed (all p>0.05). *p<0.05 **p<0.01 ***p<0.001 ****p<0.0001 #p<0.00001
